# Development and Validation of An Interpretable Machine Learning-Based Prediction Model of Postpartum Hemorrhage in Placenta Previa Following Cesarean Section: A Multicenter Study

**DOI:** 10.1007/s43032-025-01937-0

**Published:** 2025-08-12

**Authors:** Mianmian Li, Xinhui Su, Wenxin Liao, Li Huang, Yihong Yang, Xizi Wu, Yao Fan, Jing Liu, Xin Yang, Zhen Zeng, Wencheng Ding, Wanjiang Zeng, Xiaoyan Xu

**Affiliations:** Department of Obstetrics and Gynecology, Tongji Hospital, Tongji Medical College, Huazhong University of Science and Technology, Wuhan, 430030 Hubei China

**Keywords:** Postpartum hemorrhage, Placenta previa, Machine learning, Prediction model, Maternal health, Validation

## Abstract

The objective of this study is to predict the occurrence of postpartum hemorrhage in women with placenta previa based on machine learning. This retrospective study enrolled 845 singleton pregnant patients with placenta previa from two hospitals. They were allocated into a training cohort (n = 403), a testing cohort (n = 174), and the external validation cohort (n = 268). Univariate and multivariate regression analyses were employed to select clinical variables (p < 0.05), which were subsequently utilized to develop 11 machine learning prediction models. The area under the receiver operating characteristic curve (AUC), decision curve analysis (DCA), accuracy (ACC), sensitivity (SEN), and specificity (SPE) were used to evaluate the performance of the models. Besides, SHapley Additive exPlanations (SHAP) was used to interpret the role and effectiveness of variables in the predictive model. Three machine learning models with the best predictive performance were combined into a Prediction Ensemble Classifier through voting. The Gradient Boosting Machine demonstrated the best predictive performance. In the validation cohort, AUC of the Gradient Boosting Machine model is 0.810(95% CI 0.754–0.865), ACC was 0.765(95% CI 0.716–0.813), SEN was 0.613(95% CI 0.513–0.723), while these values of the Prediction Ensemble Classifier were 0.813(0.756–0.871), 0.806(0.757–0.854), and 0.480(0.375–0.597), respectively. The importance of SHAP variables in the model, ranked from high to low, is as follows: d-dimer, ultrasound diagnosis of placenta accreta spectrum, neutrophils, prothrombin time, and platelets. The Gradient Boosting Machine model demonstrated excellent performance in predicting postpartum hemorrhage in cases of placenta previa. Furthermore, SHAP analysis enabled interpretation of the variables in the model.

**Supplementary Information:**

The online version contains supplementary material available at 10.1007/s43032-025-01937-0.

## Introduction

Postpartum hemorrhage (PPH), characterized as more than 500 mL after vaginal delivery or 1000 mL after cesarean section, remains one of the most significant leading causes to maternal morbidity and mortality globally, representing roughly 27% of maternal deaths globally [1, 2]. Placenta previa (PP), a situation where the placenta partially or completely obstructs the endocervix, often accompanied by placenta accreta spectrum (PAS), is more likely to cause PPH [3–5]. Despite advances in obstetric care, accurately predicting which patients with PP are more likely to develop PPH remains a clinical challenge [6, 7], emphasizing the demand for improved risk prediction tools [8, 9].

Over the past few years, machine learning (ML) has risen as a revolutionary advancement in healthcare and disease prediction [10, 11], achieving remarkable success in predicting various obstetric outcomes, including preeclampsia, fetal growth restriction, and preterm birth [12, 13]. However, the application of ML in predicting PPH among women with PP has not yet been thoroughly explored. Previous studies have identified several risk factors for PPH, including maternal age, parity, prior cesarean delivery, and the degree of abnormal placental invasion [14–17]. However, these factors alone often fail to provide sufficient predictive accuracy due to the complex interplay of variables involved. ML algorithms, with their capacity to process complex data and detect non-linear connections, offer a novel approach to integrating these risk factors into a comprehensive framework to predict PPH in PP [18–21].

However, ML models are often criticized for their lack of interpretability. SHapley Additive exPlanations (SHAP), a concept derived from cooperative game theory that fairly allocates gains or losses among collaborators, is an advanced interpretable ML framework designed to provide in-depth explanations for predictions generated by any machine learning model [22]. In ML, the contribution of each feature to the prediction model can be quantified by calculating its SHAP value, which makes it possible to understand the influence of each feature on the prediction model [23].

Currently, research on developing interpretable ML prediction models for PPH in PP remains limited. Therefore, this study utilizes multicenter clinical data from patients with PP to develop multiple ML models for predicting PPH. These models aim to better identify patients at higher risk of PPH, thus reducing maternal morbidity and mortality, and ultimately improving patient outcomes. Additionally, SHAP values were calculated to interpret the contribution of each variable during the model development process.

## Methods

### Ethics Statement and Study Population

This research received approval from the ethics review committee's institutional review board at Tongji Hospital, Tongji Medical College, Huazhong University of Science and Technology, with an exemption from obtaining informed consent (approval number TJ-IRB202406047). All procedures involving human participants in the study were conducted following the ethical standards of the institutional and national research committees, as well as the 1964 Declaration of Helsinki and its subsequent amendments or comparable ethical standards.

**Fig. 1:**
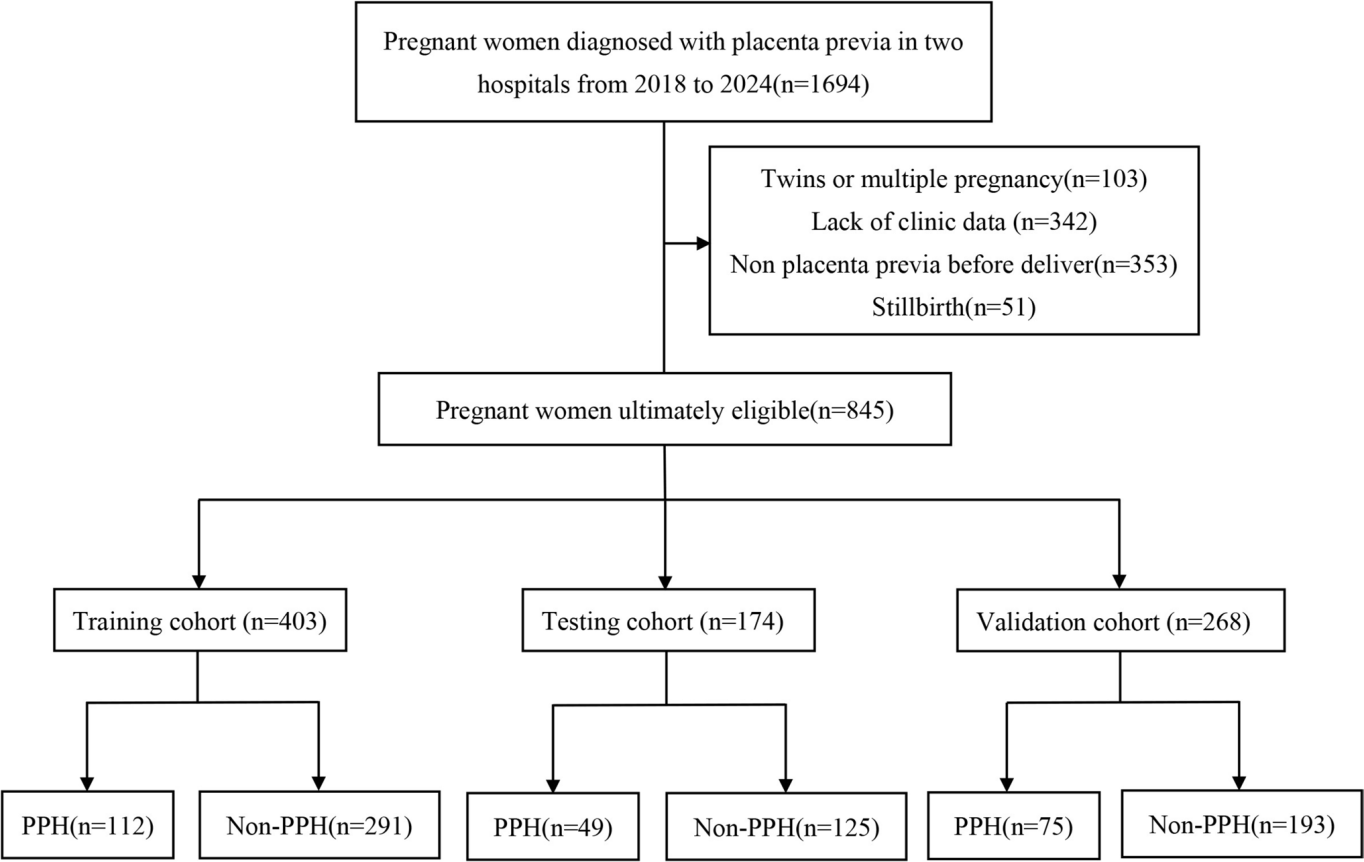
Flowchart of patient enrollment. *PPH* postpartum hemorrhage

This study was conducted at two tertiary medical centers. A total of 845 pregnant women with ultrasound-confirmed PP after 28 weeks of gestation (confirmed gestational age) who delivered between January 2018 and December 2024 were recruited, and their clinical data were collected. Among them, 577 women came from Tongji Hospital, Huazhong University of Science and Technology and they were allocated into a training cohort (n = 403) and a testing cohort (n = 174) at a 7:3 ratio [24, 25]. An additional 268 patients from Hubei Maternal and Child Health Hospital were included as an independent external validation cohort (Table 1). All the pregnant women were delivered via cesarean section. The exclusion criteria for this study are as follows: (1) twins or multiple pregnancy, (2) lack of clinical data, (3) non-placenta previa before delivery, (4) stillbirth (Fig. 1). All patients who did not meet the exclusion criteria were included in the final analysis. The flowchart of the patients' enrollment is shown in Fig 1.

**Table 1:** The clinical characteristics of pregnant women in the Training, Testing and Validation cohorts

Characteristics	Training Cohort (n = 403)		Testing Cohort (n = 174)		Validation Cohort (n = 268)		P value
	Non PPH (n = 291)	PPH (n = 112)	Non PPH (n = 125)	PPH (n = 49)	Non PPH (n = 193)	PPH (n = 75)	(P < 0.01)
Maternal age, years	32.14 ± 4.78	33.52 ± 4.52	32.06 ± 4.32	32.84 ± 3.98	32.60 ± 4.31	33.51 ± 3.98	0.398
Gravidity	2.56 ± 1.53	3.33 ± 1.78	2.62 ± 1.60	3.31 ± 1.83	3.08 ± 1.73	3.84 ± 1.88	0.000*
Parity	0.54 ± 0.62	0.97 ± 0.72	0.65 ± 0.69	0.84 ± 0.72	0.67 ± 0.63	1.01 ± 0.73	0.117
Abortions	1 ± 1.18	1.36 ± 1.45	0.94 ± 1.20	1.49 ± 1.42	1.41 ± 1.41	1.80 ± 1.59	0.000*
Cesarean sections	0.35 ± 0.54	0.85 ± 0.73	0.45 ± 0.59	0.78 ± 0.74	0.46 ± 0.61	0.89 ± 0.78	0.176
Uterine surgeries	0.35 ± 0.55	0.86 ± 0.76	0.45 ± 0.63	0.80 ± 0.74	0.41 ± 0.59	0.72 ± 0.76	0.634
D-Dimer	2.13 ± 2.41	6.13 ± 6.43	2.24 ± 2.70	5.87 ± 6.14	1.80 ± 1.10	4.50 ± 3.87	0.015
Prothrombin time, s	12.67 ± 0.91	13.48 ± 1.29	12.62 ± 1.11	13.55 ± 1.34	10.74 ± 0.66	11.37 ± 3.34	0.000*
APTT, s	33.62 ± 3.43	35.22 ± 6.27	33.90 ± 3.74	35.69 ± 3.73	26.58 ± 2.20	27.27 ± 3.29	0.000*
AST, U/L	17.78 ± 9.12	21.46 ± 14.98	20.56 ± 17.80	18.89 ± 7.52	16.42 ± 5.05	15.76 ± 4.42	0.000*
LDH, U/L	195.36 ± 48.79	230.80 ± 86.55	198 ± 50.93	223.20 ± 76.98	194.84 ± 27.68	199.27 ± 42.57	0.018
Platelet, 10⁹/L	206.13 ± 55.57	180.09 ± 51.17	207.30 ± 51.60	190.59 ± 48.59	222.21 ± 116.37	193.15 ± 51.79	0.030
Neutrophil, 10⁹/L	8.37 ± 3.26	11.03 ± 4.25	8.53 ± 3.61	12.01 ± 4.00	7.82 ± 2.55	7.40 ± 2.60	0.000*
Neutrophil to lymphocyte ratio	6.41 ± 3.99	9.56 ± 6.31	6.10 ± 3.44	10.13 ± 5.99	5.73 ± 3.24	6.28 ± 4.39	0.000*
Prenatal weight, kg	68.29 ± 8.85	71.33 ± 9.67	68.29 ± 9.53	69.26 ± 10.36	67.56 ± 8.21	68.16 ± 8.80	0.144
Prenatal BMI, kg/m^2^	26.08 ± 3.17	27.22 ± 2.99	25.92 ± 3.42	26.37 ± 3.24	26.27 ± 2.98	26.47 ± 3.12	0.468
Pre-pregnancy weight, kg	55.87 ± 8.12	58.84 ± 8.12	56.04 ± 9.05	56.12 ± 12.35	55.24 ± 7.21	55.61 ± 8.03	0.136
Pre-pregnancy BMI, kg/m^2^	21.35 ± 3.06	22.46 ± 3.25	21.28 ± 3.34	21.35 ± 4.27	21.48 ± 2.69	21.31 ± 3.79	0.416
Gestational age, weeks	36.47 ± 1.67	35.46 ± 2.36	36.31 ± 1.88	35.41 ± 1.93	34.90 ± 2.33	34.37 ± 2.75	0.000*
Blood loss, ml	435.91 ± 190.66	2132.14 ± 1451.90	443.20 ± 190.11	2114.29 ± 1408.31	481.09 ± 141.85	1416.00 ± 543.27	0.011
Assisted reproduction							0.001*
Yes	74(25%)	21(19%)	25(20%)	9(18%)	23(12%)	9(12%)	
No	217(75%)	91(81%)	100(80%)	40(82%)	170(88%)	66(88%)	
Scarred uterus							0.339
Yes	90(31%)	75(67%)	50(40%)	29(59%)	75(39%)	49(65%)	
No	201(69%)	37(33%)	75(60%)	20(41%)	118(61%)	26(35%)	
Gestational hypertension							0.161
Yes	7(2%)	5(4%)	8(6%)	3(6%)	8(4%)	2(3%)	
No	284(98%)	107(96%)	117(94%)	46(94%)	185(96%)	73(97%)	
Gestational diabetes mellitus							0.103
Yes	63(22%)	12(11%)	25(20%)	10(20%)	51(26%)	17(23%)	
No	228(78%)	100(89%)	100(80%)	39(80%)	142(74%)	58(77%)	
Placenta previa history							0.229
Yes	7(2%)	11(10%)	9(7%)	4(8%)	4(2%)	7(9%)	
No	284(98%)	101(90%)	116(93%)	45(92%)	189(98%)	68(91%)	
History of PPH							0.134
Yes	4(1%)	9(8%)	6(5%)	4(8%)	3(2%)	3(4%)	
No	287(99%)	103(92%)	119(95%)	45(92%)	190(98%)	72(96%)	
Ultrasound diagnosis of PAS							0.000*
None	149(51%)	24(22%)	65(52%)	9(19%)	54(28%)	2(3%)	
Placenta accrete	63(22%)	11(10%)	22(18%)	7(14%)	63(33%)	10(13%)	
Placenta increta	78(27%)	71(63%)	38(30%)	32(65%)	62(32%)	37(49%)	
Placenta percreta	1(0%)	6(5%)	0(0%)	1(2%)	14(7%)	26(35%)	

*PPH* Postpartum hemorrhage; *PAS* placenta accreta spectrum; *APTT* Activated partial thromboplastin time; *AST* Aspartate aminotransferase; *LDH* Lactate dehydrogenase; *BMI* body mass index. The values are presented in the form of mean ± standard deviation and percentage. “Prenatal”denotes 48 h before delivery; “Pre-pregnancy” refers to “before pregnancy”

### Variable Selection and Model Construction

Prenatal variables were extracted from electronic medical records, encompassing demographic details, medical, obstetric, surgical, family background, ultrasound diagnosis, delivery-related data, and lab results. Among them, all blood samples were collected within one week before cesarean delivery. PPH was characterized as blood loss exceeding 1000 mL after cesarean section according to the definition of the World Health Organization [1, 2].

To enhance model convergence and performance, numerical variables were standardized using z-score normalization. Variables with missing rates exceeding 20% were excluded, while missing values in the remaining variables were imputed during the data preprocessing. Univariate analysis was initially conducted to identify features with a p-value < 0.05, followed by multivariate analysis to select variables for the prediction model with a p-value < 0.05. Based on the final selected variables, 11 ML models, including 1. Logistic Regression (LR), 2. NaiveBayes, 3. Support Vector Machine (SVM), 4. K-nearest Neighbors (KNN), 5. Randomorest, 6. Extremely randomized Trees (ExtraTrees), 7. eXtreme Bradient Boosting (XGBoost), 8. LightGradient Boosting Machine (LightGBM), 9. GradientBoosting, 10. Adaptive Boosting (AdaBoost), 11. Multilayer Perceptron (MLP) were established to predict PPH in PP. The specific model establishment was performed using Python 3.7.1 (https://www.python.org) and R software 3.4.1 (http://www.Rproject.org) [26].

### Ensemble of Prediction Models

To integrate the performance of multiple models and effectively enhance predictive capability, we selected the top three performing models (MLP, SVM, and GradientBoosting) from 11 ML algorithms to construct an ensemble model named Prediction Ensemble Classifier (PEC) [27, 28]. Specifically, the predictions from each sub-model were aggregated through weighted voting, with manually assigned weights of 0.4, 0.2, and 0.4, respectively. The ensemble model establishment was performed using Python 3.7.1 (https://www.python.org).

### The Evaluation and Comparison of Models

To evaluate the performance of the model, various well-known metrics were utilized, including the receiver operating characteristic curve (ROC), and area under the curve (AUC), decision curve analysis (DCA), accuracy (ACC), sensitivity (SEN), specificity (SPE), precision (PRE) and F1 score [29]. ROC curve: The ROC curve is a graphical plot with the true positive rate (TPR) on the vertical axis and the false positive rate (FPR) on the horizontal axis. Among them, TP denotes true positives, TN denotes true negatives, FP denotes false positives, and FN denotes false negatives [21, 30, 31]. The specific metric calculations were performed using Python 3.7.1 (https://www.python.org).

- 1. AUC: AUC is employed to evaluate the capability of the model to differentiate between positive and negative instances.
  $$\begin{array}{c}\text{TPR}= (\text{TP}) /(\text{TP}+\text{ FN})\\ \text{FPR}=(\text{FP}) /(\text{FP}+\text{ TN})\end{array}$$
- 2. ACC: Accuracy represents the ratio of correctly predicted instances by the model to the total number of instances.
  $$\text{ACC}=(\text{TP}+\text{TN})/(\text{TP}+\text{TN}+\text{FP}+\text{ FN})$$
- 3. SEN or Recall: Sensitivity (also called recall) refers to the ratio of true positives predicted by the model to the total number of actual positive samples.
  $$\text{SEN}=\text{ TP}/(\text{TP}+\text{FN})$$
- 4. SPE: Specificity refers to the ratio of true negatives predicted by the model to the total number of actual negative samples.
  $$\text{SPE}=\text{TN}/(\text{TN}+\text{FP})$$
- 5. PRE: Precision quantifies the ratio of accurately predicted positive cases (true positives) to the total number of instances classified as positive (true positives plus false positives). It indicates the model's capability to minimize incorrect positive predictions.
  $$\text{PRE}=(\text{TP})/(\text{TP}+\text{FP})$$
- 6. F1 Score: The F1 score is calculated as the harmonic mean of precision and recall, serving as a unified metric that equally weighs both measures. It is particularly beneficial when dealing with uneven class distributions.
  $$\text{F}1\text{ Score}=2*\text{PRE}*\text{Recall}/(\text{PRE}+\text{Recall})$$

### Model Interpretation

The SHAP algorithm was employed to improve the interpretability of our clinical prediction model [32, 33]. The"shapviz"is an R package designed for interpreting ML model predictions. SHAP values were used to quantify the contribution of each feature to individual model predictions. The summary plot is a commonly used visualization method in SHAP, designed to display the importance of features and the way they affect the outcome. It combines feature importance with feature effect plots, illustrating the distribution of SHAP values of each feature. The feature heatmap of PEC is plotted based on the average SHAP value of the models. The calculation of SHAP values, the generation of summary plots, and heatmaps were all implemented using R software 3.4.1 (http://www.Rproject.org) [34].

### Statistical Analysis

Independent sample t-test (for normally distributed data) or Mann–Whitney U test (for non-normally distributed data) was utilized to analyze differences in continuous variables between two groups, while the χ^2^ test was employed to assess differences in proportions or rates between two groups. One-way ANOVA is used to determine whether there are statistically significant differences in the means among three or more groups. For comparing multiple proportions or rates, the Chi-square test and Fisher's exact test are employed. Quantitative variables were expressed as mean ± standard deviation (SD), and categorical variables were presented as frequency. P < 0.01 indicates a statistically significant difference [35, 36]. The flowchart of the study is illustrated in Online Resource 1.

## Results

### Clinical Baseline Characteristics

The study population comprised 845 women with PP who underwent cesarean delivery. Among them, 28% (n = 236) experienced PPH, defined as blood loss ≥ 1000 mL. As shown in Tables 1 and 2, the training cohort included 403 individuals, with 28% (n = 112) in the PPH group; the testing cohort included 174 individuals, with 28% (n = 49) in the PPH group; and the validation cohort included 268 individuals, with 28% (n = 75) in the PPH group.

**Table 2:** The clinical characteristics of pregnant women in the PPH and non-PPH group

Characteristics	PPH(n = 236)	Non-PPH(n = 609)	P value (P ≤ 0.01)
Maternal age, years	33.37 ± 4.24	32.27 ± 4.54	0.001*
Gravidity	3.49 ± 1.83	2.74 ± 1.62	0.000*
Parity	0.96 ± 0.72	0.60 ± 0.64	0.000*
Abortions	1.53 ± 1.50	1.12 ± 1.27	0.000*
Cesarean sections	0.85 ± 0.75	0.40 ± 0.58	0.000*
Uterine surgeries	0.80 ± 0.77	0.39 ± 0.58	0.000*
D-Dimer	5.55 ± 5.70	2.05 ± 2.16	0.000*
Prothrombin time, s	12.82 ± 2.38	12.05 ± 1.26	0.000*
APTT, s	32.79 ± 6.25	31.45 ± 4.59	0.003*
AST, U/L	19.12 ± 11.39	17.92 ± 10.70	0.164
LDH, U/L	219.20 ± 74.25	195.93 ± 43.69	0.000*
Platelet, 10⁹/L	186.39 ± 50.99	211.47 ± 79.69	0.000*
Neutrophils, 10⁹/L	10.08 ± 4.18	8.23 ± 3.14	0.000*
Neutrophil to lymphocyte ratio	8.64 ± 5.90	6.13 ± 3.66	0.000*
Prenatal weight, kg	69.90 ± 9.62	68.05 ± 8.80	0.008*
Prenatal BMI, kg/m^2^	26.81 ± 3.09	26.10 ± 3.16	0.004
Pre-pregnancy weight, kg	57.26 ± 9.97	55.71 ± 8.04	0.034
Pre-pregnancy BMI, kg/m^2^	21.86 ± 3.68	21.38 + 3.00	0.051
Gestational age, weeks	35.14 ± 2.45	35.94 ± 2.07	0.000*
Blood loss, ml	451.72 ± 177.41	1900.85 ± 1266.40	0.000*
Assisted reproductive technology			0.244
Yes	39 (17%)	122 (20%)	
No	197 (83%)	487 (80%)	
Scarred uterus			0.000*
Yes	153 (65%)	215 (35%)	
No	83 (35%)	395 (65%)	
Gestational hypertension			0.756
Yes	10 (4%)	23 (4%)	
No	226 (96%)	586 (96%)	
Gestational diabetes			0.044
Yes	39 (17%)	139 (23%)	
No	197 (83%)	470 (77%)	
Placenta previa history			0.000*
Yes	22 (9%)	20 (3%)	
No	214 (91%)	589 (97%)	
History of postpartum hemorrhage			0.001*
Yes	16 (7%)	13 (2%)	
No	220 (93%)	596 (98%)	
Ultrasound diagnosis of PAS			0.000*
None	35 (15%)	268 (44%)	
Placenta accreta	28 (12%)	148 (24%)	
Placenta increta	140 (59%)	178 (29%)	
Placenta percreta	33 (14%)	15 (2%)	

*PPH* Postpartum hemorrhage; *PAS* placenta accreta spectrum; *APTT* Activated partial thromboplastin time; *AST* Aspartate aminotransferase; *LDH* Lactate dehydrogenase; *BMI* body mass index. The values are presented in the form of mean ± standard deviation and percentage. “Prenatal”denotes 48 h before delivery; “Pre-pregnancy” refers to “before pregnancy”

Baseline characteristics revealed significant differences between the PPH and non-PPH groups. Women with PPH had a higher prevalence of risk factors, including maternal age, gravidity, parity, abortions, cesarean sections, gestational age, d-dimer, platelet, prothrombin time (PT), neutrophils, neutrophil to lymphocyte ratio, and prenatal weight (p < 0.01). Additionally, the PPH group exhibited a higher proportion compared to the non-PPH group, like ultrasound diagnosis of PAS (85% vs. 56%, p < 0.01). No significant differences were observed in gestational diabetes, gestational hypertension, assisted reproductive technology**,** prenatal body mass index (BMI), or pre-pregnancy BMI between the two groups (p > 0.01) (Table 2).

### Screening of Clinical Variables

Five clinical variables were selected for model establishment, including ultrasound diagnosis of PAS, elevated d-dimer levels, elevated platelet count, prolonged PT, and increased neutrophil. The process of the variables ultimately selected is shown in Online Resource 2***.*** The selected variables and specific statistical results are shown in Table 3. According to the odds ratio (OR) values (Table 3), while controlling for other variables, it was found that four variables have a promoting effect on the outcome of PPH, including ultrasound diagnosis of PAS, elevated d-dimer levels, prolonged PT, and increased neutrophil, while elevated platelet count has a negative effect on the outcome of PPH.

**Table 3:** Clinical variables with a P-value < 0.05 were selected through regression analysis

Variables	P-value	OR	OR (95%CI)
Ultrasound diagnosis of PAS	0.000*	1.918	1.408–2.614
D-Dimer	0.000*	2.045	1.381–3.028
Platelet count (PLT)	0.033*	0.709	0.516–0.974
Prothrombin time (PT)	0.044*	1.466	1.011–2.128
Neutrophils	0.011*	1.723	1.132–2.623

*95%CI* 95% confidence interval (lower and upper limits); *PAS* placenta accreta spectrum; *OR* odds ratio

### Model Merits

**Fig. 2:**
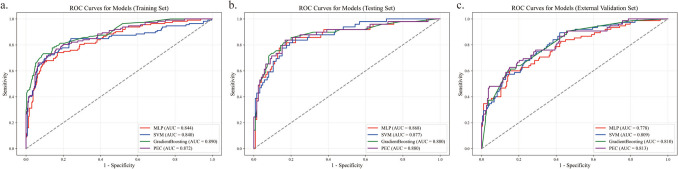
ROC curves of the three prediction models and the resemble classifier. (**a**) The ROC curves of the models in training cohort. (**b**) The ROC curves of the models in testing cohort. (**c**) The ROC curves of the models in validation cohort. *ROC* the receiver operating characteristic curve; *PEC* Prediction ensemble classifier; *SVM* Support vector machine; *MLP* Multilayer perceptron

**Fig. 3:**
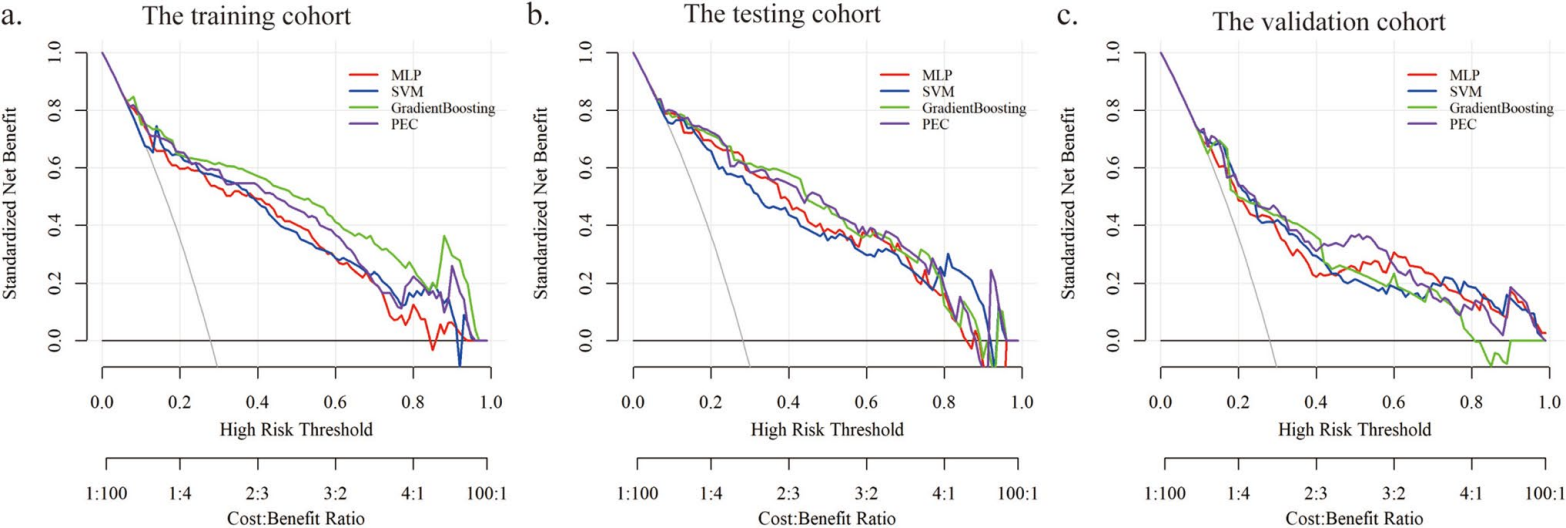
DCA curves of the three prediction models and the resemble classifier. The x-axis represents the threshold probability, and the y-axis represents the net benefit. The gray line represents the assumption that all patients have PPH, and the black line represents the assumption that no patients have PPH. (**a**) The DCA curves of the models in training cohort. (**b**) The DCA curves of the models in testing cohort. (**c**) The DCA curves of the models in validation cohort. *DCA* decision curve analysis; *PEC* Prediction ensemble classifier; *SVM* Support vector machine; *MLP* Multilayer perceptron

Ultimately, 11 machine learning prediction models were developed based on the clinical data of patients, and the performance of these models was summarized in Online Resource 3. Among the 11 machine learning models, MLP, SVM, and GradientBoosting ML models demonstrated superior performance. Besides, the performance of the ensemble model PEC was almost the same as the GradientBoosting model. In the testing cohort, the AUCs of MLP, SVM, GradientBoosting, and PEC models were 0.868(95%CI 0.803–0.933), 0.877(95%CI 0.820–0.933), 0.880(95%CI 0.816–0.943), and 0.880(95%CI 0.817–0.942) (Table 4 and Fig. 2b). In the external validation cohort, the AUCs of MLP, SVM, GradientBoosting, and PEC models was 0.778(95%CI 0.715–0.842), 0.809(95%CI 0.752–0.865), 0.810(95%CI 0.754–0.865), and 0.813(95%CI 0.756–0.871), respectively (Table 4 and Fig. 2c). Decision curve analysis showed that all four models provided a high overall net benefit, which was more advantageous than the strategies of treating all or none (Fig. 3).

**Table 4:** Performances of the models in the training, testing and validation cohorts

Cohort	Model name	AUC (95%CI)	ACC (95%CI)	SEN (95%CI)	SPE (95%CI)	PRE (95%CI)
Training cohort	MLP	0.844(0.800–0.889)	0.806(0.769–0.846)	0.696(0.603–0.779)	0.849(0.808–0.889)	0.639(0.558–0.721)
	SVM	0.840(0.788–0.891)	0.814(0.777–0.851)	0.741(0.655–0.818)	0.842(0.799–0.885)	0.643(0.562–0.725)
	GradientBoosting	0.890(0.854–0.927)	0.816(0.777–0.854)	0.813(0.744–0.861)	0.818(0.771–0.861)	0.632(0.557–0.715)
	PEC	0.872(0.832–0.912)	0.821(0.784–0.856)	0.732(0.655–0.809)	0.856(0.812–0.896)	0.661(0.586–0.744)
Testing cohort	MLP	0.868(0.803–0.933)	0.839(0.782–0.891)	0.796(0.686–0.900)	0.856(0.792–0.913)	0.684(0.571–0.804)
	SVM	0.877(0.820–0.933)	0.805(0.741–0.856)	0.837(0.727–0.927)	0.792(0.725–0.858)	0.612(0.492–0.729)
	GradientBoosting	0.880(0.816–0.943)	0.816(0.759–0.874)	0.857(0.755–0.943)	0.800(0.725–0.872)	0.627(0.508–0.740)
	PEC	0.880(0.817–0.942)	0.833(0.776–0.891)	0.837(0.729–0.932)	0.832(0.760–0.892)	0.661(0.534–0.773)
Validation cohort	MLP	0.778(0.715–0.842)	0.795(0.743–0.840)	0.293(0.195–0.405)	0.990(0.974–1.000)	0.917(0.792–1.000)
	SVM	0.809(0.752–0.865)	0.724(0.672–0.776)	0.720(0.618–0.822)	0.725(0.665–0.786)	0.505(0.412–0.598)
	GradientBoosting	0.810(0.754–0.865)	0.765(0.716–0.813)	0.613(0.513–0.723)	0.824(0.767–0.878)	0.575(0.461–0.675)
	PEC	0.813(0.756–0.871)	0.806(0.757–0.854)	0.480(0.375–0.597)	0.933(0.897–0.965)	0.735(0.604–0.848)

*AUC* area under the receiver operating characteristic curve; *ACC* accuracy; *SEN* sensitivity; *SPE* specificity; *CI* Confidence interval; *PEC* Prediction ensemble classifier; *SVM* Support vector machine; *MLP* Multilayer perceptron

### Comparison of the Three Models

The AUC of the GradientBoosting was higher than that of MLP and SVM in the training, testing, and external validation cohorts. The AUC of the GradientBoosting was higher than that of PEC in the training, testing cohorts and almost the same as PEC model in the external validation cohorts. In the testing cohort, the ACC, SPE, and PRE of MLP were higher than those of SVM and GradientBoosting, while the SEN of GradientBoosting was higher than that of MLP and SVM. In the external validation cohort, the SEN of SVM was higher than that of MLP and GradientBoosting, while the SPE and PRE of MLP were higher than those of SVM and GradientBoosting (Table 4 and Fig. 2).

### The Interpretation of the Models

In the*** s***ummary plot in SHAP, the horizontal axis shows the impact of each feature on the prediction outcome. Points located further from the center signify a stronger influence of the feature on the predictions of the model. The vertical axis indicates the order of feature values from highest to lowest based on their overall contribution to the predictions of the model, with the top features exerting the most significant influence on the output of the model. The influence and direction of features vary across different prediction models.

**Fig. 4:**
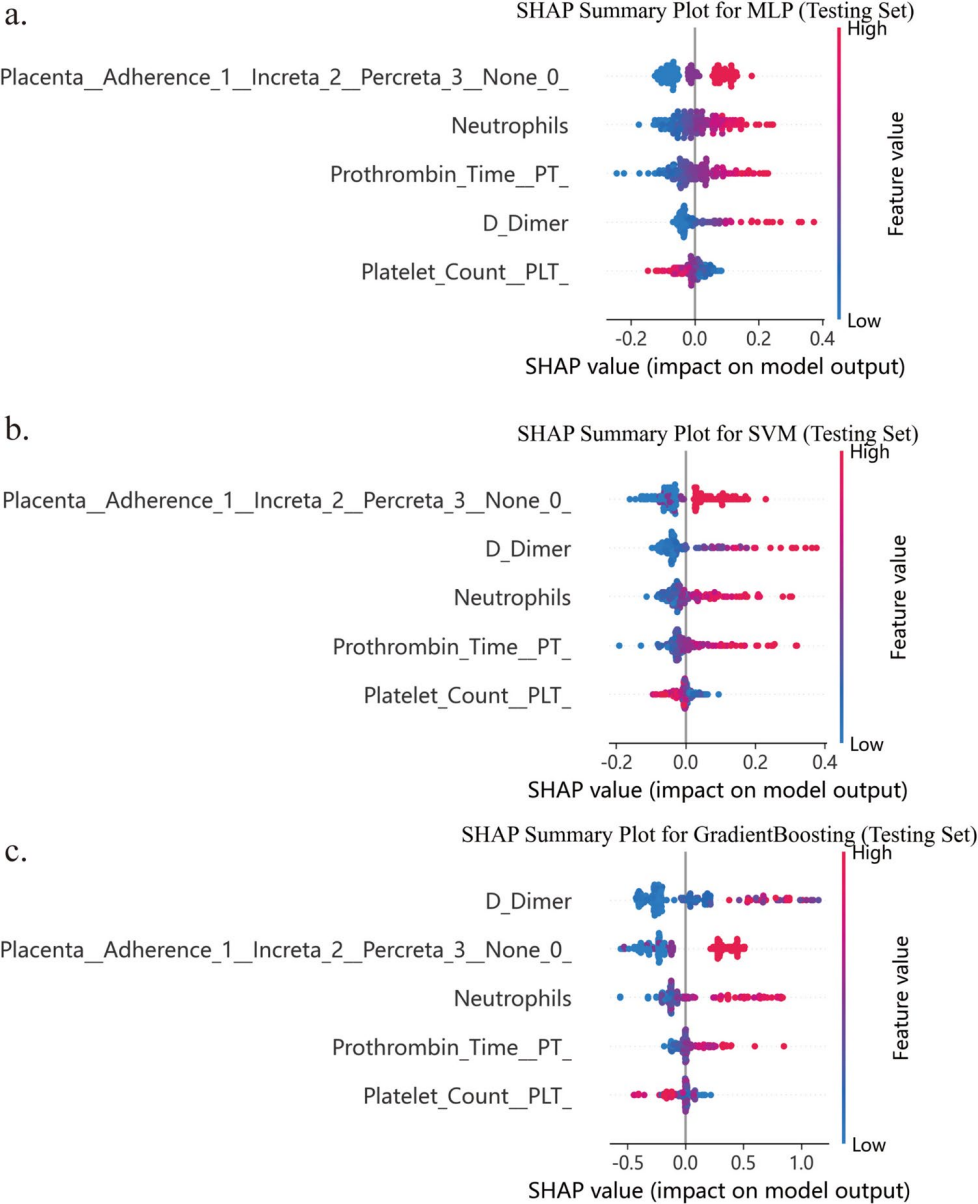
Shap summary plots for the three prediction models. Each point corresponds to the feature value of a sample (red for higher values, blue for lower values). (**a)** Shap summary plots of MLP model. (**b**) Shap summary plots of SVM model. (**c**) Shap summary plots of GradientBoosting model. *SVM* Support vector machine; *MLP* Multilayer perceptron

**Fig. 5:**
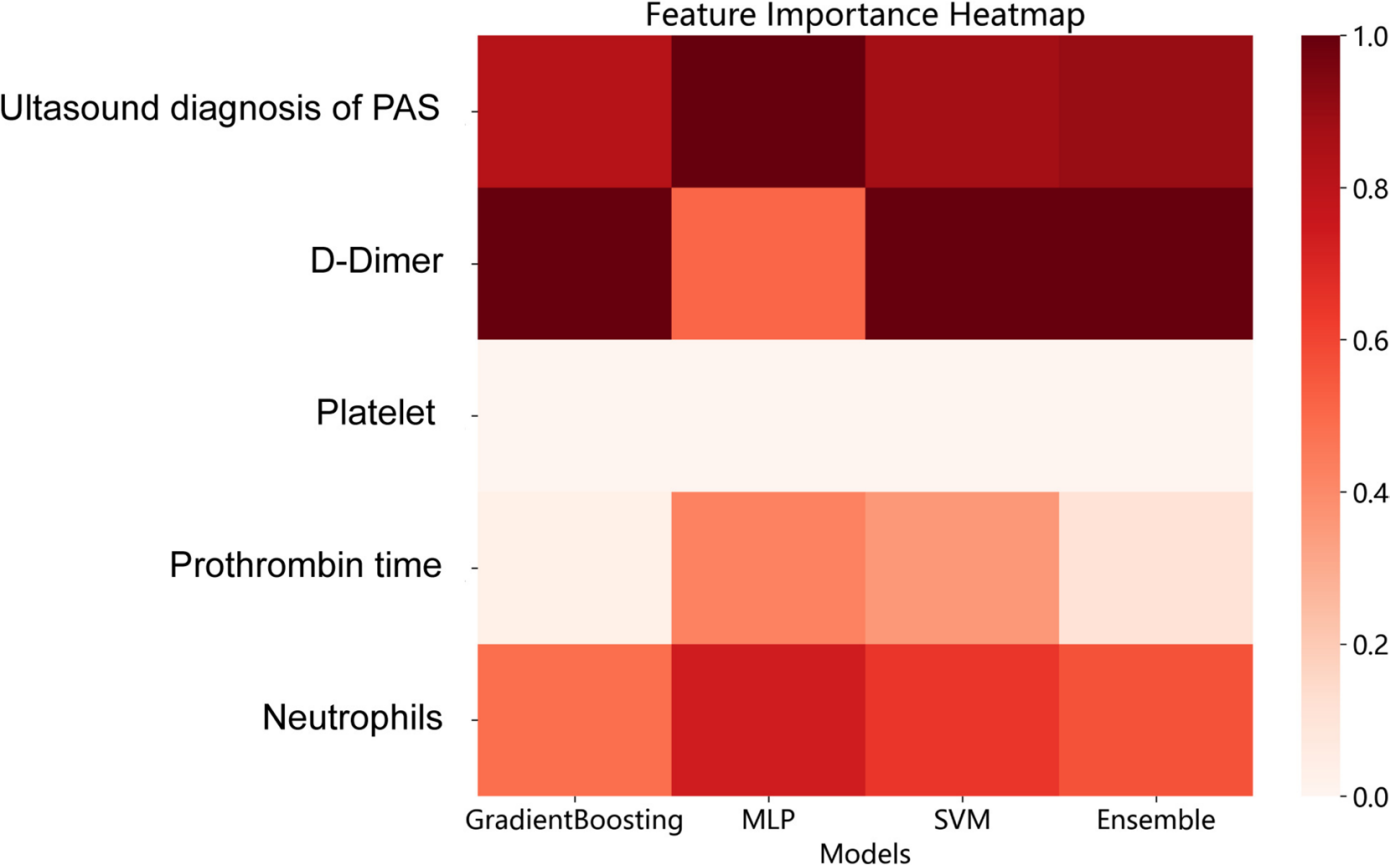
The feature importance heatmap of the models. *PEC* Prediction ensemble classifier; *SVM* Support vector machine; *MLP* Multilayer perceptron

As shown in the Fig. 4, the order of feature values from highest to lowest in the MLP model were ultrasound diagnosis of PAS, neutrophils, PT, d-dimer and platelet (Fig. 4a). The order of features from highest to lowest in the SVM model was ultrasound diagnosis of PAS, d-dimer, neutrophils, PT, and platelet (Fig. 4b). And the order of features from highest to lowest in the GradientBoosting model was d-dimer, ultrasound diagnosis of PAS, neutrophils, PT, and platelet (Fig. 4c). Besides, the positive SHAP values of platelet indicate a negative effect on the prediction outcome in these three models. As shown in the feature heatmap, the order of features from highest to lowest in the PEC model was d-dimer, ultrasound diagnosis of PAS, neutrophils, PT, and platelet (Fig. 5).

## Discussion

This study identified five key predictive variables for PPH through univariate and multivariate regression analysis and ranked them by SHAP values. Interestingly, several previously recognized risk factors for PPH, such as parity, abortions, and a prior occurrence of PPH, were excluded in the final model, which suggests that these factors may not contribute to PPH risk in PP to the extent previously assumed [37, 38]. Based on these variables, we developed and validated three interpretable machine learning prediction models of PPH in PP following cesarean section. Among these three selected ML models, the GradientBoosting model demonstrated the best performance with AUCs of 0.890 in the training cohort, 0.880 in the testing cohort, and 0.810 in the external validation cohort. The actual contribution of the variables to the GradientBoosting model ranked from high to low was d-dimer, ultrasound diagnosis of PAS, neutrophils, PT, and platelet. Based on these findings, clinicians can early identify high-risk pregnant women earlier and arrange deliveries in well-equipped comprehensive hospitals. Furthermore, the GradientBoosting model can generate the patient's probability of PPH by inputting these five predictive variables, thereby assisting clinicians in risk assessment.

PPH continues to be a major contributor to maternal morbidity and mortality, and its prediction continues to pose challenges[39, 40]. Japanese scholars utilized 11 clinical variables to construct five ML models, and a deep learning model composed of a two-layer neural network to predict the risk of PPH in women undergoing vaginal delivery. However, the predictive performance of the models was limited and requires further validation with large datasets [41]. Jill M. Westcott and their team in New York utilized ML techniques to identify patients at risk for PPH during obstetric delivery. Their model demonstrated performance nearly comparable to models using more comprehensive datasets, highlighting its potential utility in clinical applications [42]. Similarly, Baoxia Cui and her team developed a prediction model deployed in a web-based application, capable of forecasting the risk of intraoperative hemorrhage during cesarean scar ectopic pregnancy (CSEP) surgery [43]. These machine learning methods have positive significance for predicting PPH, but the effect of specific variables on the outcome is not clear. This study has made innovations in this aspect. In addition to these studies, several international organizations have established risk prediction and warning systems for PPH. For example, the American College of Obstetricians and Gynecologists (ACOG), the California Maternal Quality Care Collaborative (CMQCC), and the Association of Women's Health, Obstetric and Neonatal Nurses (AWHONN) have developed risk assessment tools that stratify pregnant women into low, moderate, and high-risk categories for PPH [7, 44–47]. These guidelines are primarily based on traditional clinical risk factors. Some women without obvious risk factors may also experience postpartum hemorrhage. Compared to these guidelines, ML offers higher predictive accuracy and greater clinical value.

In this study, we developed interpretable ML models based on SHAP values to predict the risk of PPH in patients with PP. The model was trained and tested using internal clinical data, followed by external validation to ensure its robustness. However, there are some limitations in this study; differences in prenatal examination results across different hospitals may affect the predictive performance of the model. We could not perform further analysis due to insufficient relevant data. Future large-scale multicenter validation studies will help us develop more reliable ML models.

## Conclusions

The Gradient Boosting Machine model demonstrated excellent performance in predicting PPH in cases of PP. Furthermore, SHAP analysis enabled interpretation of the variables in the model.

## Supplementary Information

Below is the link to the electronic supplementary material.

Supplementary file1 (PDF 626 KB)

## Data Availability

The datasets used in this study are available from the corresponding author upon reasonable request.

## Abbreviations

*PPH*: Postpartum hemorrhage
*PP*: Placenta previa
*SHAP*: SHapley Additive exPlanations
*ML*: Machine learning
*ROC*: Receiver operating characteristic
*AUC*: Area under the receiver operating characteristic curve
*DCA*: Conducting decision curve analysis
*ACC*: Accuracy
*SEN*: Sensitivity
*SPE*: Specificity
*LR*: Logistic regression
*LightGBM*: Light gradient boosting machine
*MLP*: Multilayer perceptron
*PAS*: Placenta Accreta Spectrum
*PT*: Prothrombin Time
