# Supplementary material for: Development and Validation of An Interpretable Machine Learning-Based Prediction Model of Postpartum Hemorrhage in Placenta Previa Following Cesarean Section: A Multicenter Study
Source: Reprod Sci. 2025 Aug 12;32(9):3062–73. doi: 10.1007/s43032-025-01937-0 (PMC12443912; doi:10.1007/s43032-025-01937-0)
Supplement: Supplementary file 1 — Supplementary file1 (PDF 626 KB) [file 43032_2025_1937_MOESM1_ESM.pdf]

Online Resource 1: The flowchart of this study.

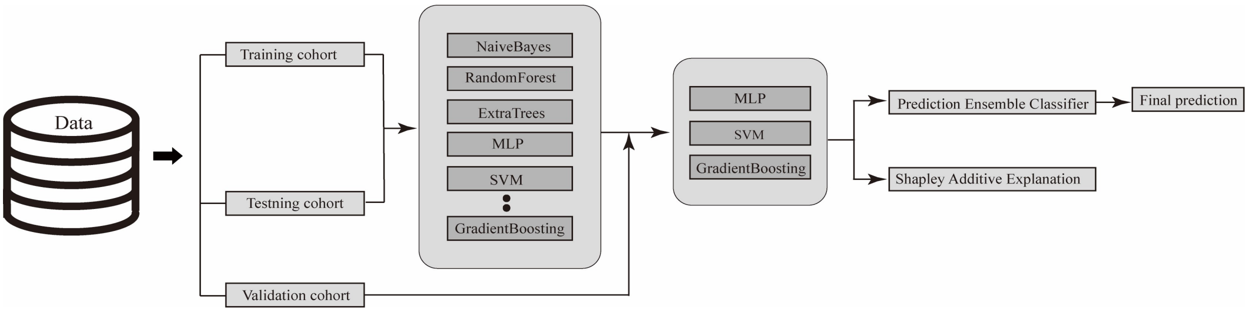

Online Resource 2: the 5 variables ultimately selected through logistic regression.

Variables	Univariate analysis (p<0.05)	Multivariate analysis (p<0.05)
Age, years	0.0099	0.650
Gravidity	0.0001	0.828
Parity	0.0000	0.866
Cesarean sections	0.0000	——
Uterine surgeries	0.0000	0.503
Scarred uterus	0.0000	0.088
Ultrasound diagnosis of PAS	0.0000	**0.000****
D-Dimer	0.0000	**0.000****
Prothrombin Time, s	0.0000	**0.044****
APTT, s	0.0036	0.404
AST, U/L	0.0088	0.624
LDH, U/L	0.0000	0.968
Platelet count, 10^9/L	0.0000	**0.034****
Neutrophils, 10^9/L	0.0000	**0.011****
Neutrophil-to-Lymphocyte ratio	0.0000	0.352
Pre-pregnancy weight, kg	0.0028	——
Prenatal weight, kg	0.0033	——
Prenatal BMI, kg/m²	0.0013	——
Pre-pregnancy BMI, kg/m²	0.0019	——
Gestational age, weeks	0.0000	0.187
Placenta previa history	0.0029	0.330
Postpartum hemorrhage history	0.0028	0.117

Abbreviations: PAS, Placenta Accreta Spectrum; APTT: Activated Partial Thromboplastin Time; AST: Aspartate Aminotransferase; LDH: Lactate Dehydrogenase; BMI, Body Mass Index. “Prenatal”denotes 48 hours before delivery; “Pre-pregnancy” refers to “before pregnancy”.

Online Resource 3: performances of the 11 ML models in the testing and validation cohorts.

Cohorts	Models	AUC (95%CI)	ACC (95%CI)	SEN (95%CI)	SPE (95%CI)	PRE (95%CI)	F1 (95%CI)
Testing cohort	LR	0.860(0.793-0.928)	0.839(0.787-0.891)	0.755(0.641-0.867)	0.872(0.810-0.929)	0.698(0.562-0.818)	0.725(0.620-0.818)
	NaiveBayes	0.835(0.762-0.909)	0.828(0.764-0.879)	0.694(0.562-0.822)	0.880(0.823-0.936)	0.694(0.561-0.820)	0.694(0.587-0.792)
	SVM	0.877(0.820-0.933)	0.805(0.747-0.862)	0.837(0.720-0.933)	0.792(0.722-0.862)	0.612(0.487-0.727)	0.707(0.600-0.796)
	KNN	0.822(0.755-0.889)	0.776(0.718-0.833)	0.714(0.583-0.836)	0.800(0.725-0.865)	0.583(0.463-0.705)	0.642(0.531-0.742)
	RandomForest	0.826(0.754-0.897)	0.799(0.736-0.856)	0.694(0.558-0.813)	0.840(0.769-0.899)	0.630(0.500-0.754)	0.660(0.543-0.763)
	ExtraTrees	0.807(0.732-0.883)	0.753(0.684-0.810)	0.714(0.587-0.844)	0.768(0.688-0.837)	0.547(0.422-0.678)	0.619(0.505-0.712)
	XGBoost	0.840(0.767-0.913)	0.816(0.758-0.874)	0.816(0.702-0.917)	0.816(0.742-0.879)	0.635(0.519-0.753)	0.714(0.612-0.800)
	LightGBM	0.852(0.782-0.922)	0.782(0.718-0.839)	0.857(0.750-0.956)	0.752(0.680-0.823)	0.575(0.458-0.687)	0.689(0.589-0.776)
	GradientBoosting	0.880(0.816-0.943)	0.816(0.759-0.868)	0.857(0.750-0.947)	0.800(0.727-0.870)	0.627(0.516-0.742)	0.724(0.624-0.807)
	AdaBoost	0.833(0.757-0.908)	0.839(0.776-0.891)	0.633(0.492-0.771)	0.920(0.872-0.962)	0.756(0.625-0.886)	0.689(0.565-0.795)
	MLP	0.868(0.803-0.933)	0.839(0.787-0.891)	0.796(0.680-0.907)	0.856(0.797-0.917)	0.684(0.559-0.800)	0.736(0.635-0.823)
Validation cohort	LR	0.807(0.747-0.867)	0.806(0.761-0.847)	0.333(0.235-0.432)	0.990(0.973-1.000)	0.926(0.808-1.000)	0.490(0.365-0.602)
	NaiveBayes	0.819(0.764-0.875)	0.799(0.746-0.843)	0.493(0.388-0.611)	0.917(0.880-0.954)	0.698(0.562-0.816)	0.578(0.465-0.667)
	SVM	0.809(0.752-0.865)	0.724(0.672-0.776)	0.720(0.618-0.821)	0.725(0.665-0.792)	0.505(0.410-0.598)	0.593(0.508-0.673)
	KNN	0.665(0.598-0.732)	0.728(0.675-0.780)	0.293(0.192-0.400)	0.896(0.852-0.938)	0.524(0.375-0.674)	0.376(0.263-0.483)
	RandomForest	0.786(0.722-0.850)	0.761(0.709-0.810)	0.520(0.407-0.632)	0.855(0.801-0.905)	0.582(0.460-0.707)	0.549(0.441-0.641)
	ExtraTrees	0.742(0.668-0.815)	0.765(0.713-0.817)	0.613(0.507-0.735)	0.824(0.769-0.876)	0.575(0.468-0.684)	0.594(0.497-0.679)
	XGBoost	0.798(0.741-0.854)	0.765(0.716-0.813)	0.427(0.312-0.541)	0.896(0.847-0.937)	0.615(0.491-0.745)	0.618(0.396-0.613)
	LightGBM	0.826(0.771-0.881)	0.784(0.731-0.832)	0.627(0.512-0.738)	0.845(0.793-0.896)	0.610(0.500-0.718)	0.594(0.504-0.704)
	GradientBoosting	0.810(0.754-0.865)	0.765(0.709-0.813)	0.613(0.500-0.720)	0.974(0.770-0.876)	0.575(0.459-0.676)	0.491(0.497-0.683)

AdaBoost	0.781(0.717-0.845)	0.799(0.746-0.847)	0.347(0.231-0.451)	0.990(0.949-0.995)	0.839(0.692-0.963)	0.444(0.373-0.607)
MLP	0.778(0.715-0.842)	0.795(0.743-0.840)	0.293(0.192-0.391)	0.933(0.974-1.000)	0.917(0.778-1.000)	0.581(0.310-0.554)

Abbreviation: ML: machine learning; CI: Confidence Interval; AUC: area under the receiver operating characteristic curve; ACC: accuracy; SEN: sensitivity; SPE: specificity; LR: Logistic Regression; SVM: Support Vector Machine; KNN: k-nearest neighbors; ExtraTrees: Extremely randomized Trees; XGBoost: eXtreme Bradient Boosting; LightGBM: LightGradient Boosting Machine; AdaBoost: adaptive boosting; MLP:Multilayer Perceptron.
